# True and Moral by Repetition: Unveiling the Impact of Exposure on Positive Stereotypes Perception

**DOI:** 10.5334/irsp.933

**Published:** 2024-08-07

**Authors:** Simone Mattavelli, Claudia Bianchi, Marco Brambilla, Matteo Motterlini

**Affiliations:** 1 University of Milano-Bicocca, Italy; 2 Vita-Salute San Raffaele University, Italy

**Keywords:** positive stereotypes, repetition, truth, truth effect, moral-repetition effect

## Abstract

Despite their apparent benevolence, positive stereotypes have negative effects on person and group perception. However, little is known about how exposure can intensify these negative consequences. In two pre-registered experiments (total N = 240) we investigated the effect of exposure on believability and moral condemnation of positive stereotypes. In Experiment 1, participants rated the truth value of positive stereotypes, which were either previously encountered or not during an exposure phase. Repeated positive stereotypes were perceived as more true than unrepeated ones, indicating a truth effect. In Experiment 2, we replicated the truth effect and further found that exposure to stereotypes reduced their moral condemnation, indicating a moral-repetition effect. Extending the truth effect and moral-repetition effect research to positive stereotypes, our findings emphasize the need to raise awareness of the impact of exposure on reinforcing the believability and moral condemnation of stereotypical beliefs.

## Introduction

Stereotypes have pervasive and profound consequences on person and group perception (Hilton & Von Hippel 1996). Classic work in social psychology has demonstrated that stereotypes lead to biases in how individuals are perceived (Tajfel & Turner 1986), how they are interacted with (Giles & Johnson 1987), and how their behaviours are interpreted (Kunda & Sinclair 1999). Moreover, stereotypes have a widespread impact, influencing information processing, self-perception, and performance (e.g., Inzlicht & Ben-Zeev 2003; Macrae & Bodenhausen 2000; Steele & Aronson 1995).

In many modern societies, social norms condemn negative stereotypical attributions about outgroup members (e.g., ‘women are bad at math’). In a socio-political climate where negative stereotypes have become inappropriate, people might compensate for avoiding negative views of outgroups by highlighting their positive attributes (Bergsieker et al. 2012). Consequently, individuals make positive yet stereotypic statements about group members (e.g., ‘women are caring’). Positive stereotypes are defined as favourable beliefs about members of social groups that imply domain-specific advantage, favourability, or superiority based on category membership (Czopp et al. 2015). Examples of positive stereotypes include statements such as ‘African Americans are naturally athletic’, ‘Mexicans are fun-loving’ and ‘Elderly people are wise’. While theories and research suggest that positive stereotypes may offer psychological benefits to target group members (Crocker & Major 1989), a growing body of literature points to their pervasive negative consequences. Positive stereotypes attributed to lower status groups often lead to associations with subordination (Ridgeway 1992). Endorsing positive stereotypes reflects a tendency to oversimplify complex groups of individuals (Czopp et al. 2015). By attributing certain positive traits to entire social categories, positive stereotypes overlook the diversity and individuality within these groups. This oversimplification can lead to the perpetuation of inaccurate beliefs and expectations, which may limit opportunities and create barriers for individuals who do not conform to the stereotype. Moreover, positive stereotypes set expectations that prescribe target’s behaviour and influence individuals’ response to it. For instance, women who promote their own competence and accomplishments tend to be professionally derogated (Rudman 1998). Positive stereotypes have also been found to negatively impact self-perception (e.g., Barreto et al. 2010), feelings (e.g., Calogero & Jost 2011), and performance (e.g., Cheryan & Bodenhausen 2000) of the stereotyped individuals.

Due to their association with traits generally perceived as favourable, advantageous, and flattering, positive stereotypes enjoy acceptability and are perpetuated across various contexts and situations, undetected by society’s antibias vigilance. While many studies have focused on the negative influence of exposure to positive stereotypes (see Czopp et al. 2015), little attention has been paid to understanding *how* exposure can exert its negative effects. In this study, our aim is to investigate the impact of exposure on (i) believability and (ii) moral condemnation of positive stereotypes. Investigating how exposure affects the believability and the moral condemnation of stereotypes has both theoretical and practical implications. Theoretically, it provides insights into the cognitive mechanisms underlying the formation and reinforcement of stereotypes. From an applied perspective, it can help media practitioners, content creators, and educators design messages that counteract stereotypes and promote accurate and positive representations of diverse social groups.

Our first hypothesis, focusing on the impact of stereotypes exposure on their believability, is rooted in the phenomenon known as ‘truth effect’ (see Unkelbach et al. 2019). The truth effect suggests that individuals perceive statements they have been exposed to as more truthful, regardless of their actual accuracy, contributing to the endorsement of claims. One theoretical explanation for this effect is that repetition enhances processing fluency (Reber & Schwarz 1999; Unkelbach 2006). Repeatedly encountered information becomes easier to process (i.e., more fluent). As fluency and subjective truth are interconnected, repetition ultimately heightens the perceived truth of statements (Fazio et al. 2015). The truth effect is a pervasive phenomenon (see Dechêne et al. 2010, for meta-analysis) observed across various domains, including social-political opinions (Arkes et al. 1989), rumours (DiFonzo et al. 2016), fake news (Pennycook et al. 2018), and conspiratorial beliefs (Béna et al. 2023). Our study tests the truth effect on positive stereotypes. In doing so, our work extends prior research on human social cognition by combining the insights from stereotyping and the truth effect. We hypothesize that participants would perceive repeated stereotypes as more truthful compared to stereotypes not encountered in the immediate past.

Related to this research question, Oğuz Taşbaş and Unkelbach (2022) found a truth effect with positive traits attributed to fictitious social groups. However, it is essential to recognize that the impact of exposure may differ when it comes to generalized beliefs about existing social groups. Evaluating the effect of repetition on the truthfulness of statements describing fictitious or previously unknown groups (e.g., ‘Tikus are friendly’) differs from establishing the same effect on deeply entrenched beliefs about well-known social groups (e.g., ‘Germans are efficient’) in several ways. Firstly, individuals may incorporate additional variables, such as their direct experiences with the target group, when assessing the truthfulness of stereotypical beliefs. Secondly, stereotypes about social groups are shared beliefs that individuals have likely encountered before. Testing the impact of repetition on top of existing knowledge adds a layer of complexity. For instance, prior research has shown that each additional repetition tends to have a diminishing effect on perceived truth (Hassan & Barber 2021). Taken together, these nuances highlight the non-obvious nature of the truth effect with stereotypes about real social groups and underscore the importance of further exploration into this research question.

Our second hypothesis, claiming that exposure to stereotypes may diminish the moral condemnation of such stereotypes, is consistent with the ‘moral-repetition effect’ (Effron 2022; Effron & Raj 2020; Pillai et al. 2023). The moral-repetition effect refers to the tendency to reduce the moral condemnation of unethical acts as exposure to such acts increases. Effron (2022) explained this phenomenon as being driven by both affective and cognitive mechanisms. In affective terms, this effect is based on the idea that feelings influence moral judgments (Greene & Haidt 2002; Haidt 2001). Whereas people experience intense negative affect when encountering a moral transgression for the first time, this intensity diminishes with repeated exposure to the same transgression. In cognitive terms, repeated encounters with unethical acts lead to a feeling of familiarity, and people may mistake familiarity for prevalence (Weaver et al. 2007). Thus, repetition might lead people to assume that a behavioural misconduct is more common, reducing its perceived ethical wrongness (Lindström et al. 2018). Based on the moral-repetition effect, we propose that the moral condemnation of positive stereotypes may be influenced by their exposure: endorsing repeatedly encountered stereotypes to form impressions about new members of a target group should be perceived as less morally wrong than endorsing newly encountered stereotypes.

In Experiment 1, we used positive stereotypes in the standard version of the truth-by-repetition paradigm (Hasher et al. 1977). This paradigm consisted of two different phases. In the exposure phase, participants were presented with a series of stereotypes. In the judgment phase, participants rated the truth value of the stereotypes encountered in the exposure phase (repeated) plus other stereotypes not encountered in the exposure phase (unrepeated). A truth effect would manifest as higher truth ratings assigned to the repeated stereotypes compared to the unrepeated ones. Experiment 2 tested the impact of repetition on both the perception of believability and the level of moral condemnation of positive stereotypes. Drawing upon the moral-repetition effect, we hypothesized that endorsing repeated stereotypes to evaluate any new member of the target group would be perceived as less morally wrong than endorsing unrepeated stereotypes.

## Transparency and Openness

The experiments were preregistered before data collection on Open Science Framework (OSF).^1^ For both the experiments, we report all measures, manipulations, and data exclusions. Targeted sample sizes were determined in advance of data collection. Verbatim materials are posted in the Supplemental Material. Data and analysis code are publicly available on OSF.^2^ The project received ethics approval from our University Committee.

## Experiment 1

In Experiment 1, we tested the truth effect of positive stereotypes. Recent research has indicated that participants are more likely to believe in and express greater liking for fictitious groups associated with repeated positive traits presented as stereotypical (Oğuz Taşbaş & Unkelbach 2022). However, it remains unknown whether the impact of repetition on truth can be replicated with existing social groups and well-established stereotypes that enjoy widespread consensus. Experiment 1 addressed this intriguing question.

### Method

We adopted a one factor (Stereotypes repetition: repeated vs. unrepeated) within-subject ANOVA design. Truth ratings (averaged among repeated vs unrepeated statements) were the outcome variable.

#### Participants

We conducted an a priori power analysis on G*Power 3.1 (Faul et al. 2009). Based on this analysis, collecting 120 participants allowed detecting an effect size as small as Cohen’s *d* = 0.30 (small to medium), in a two-tailed paired-sample t-test (difference between the average truth assigned to repeated vs. unrepeated stereotypes), at a = .05, and power 1–b = .90. We pre-registered a sequential analysis approach (Lakens 2014), with a single interim analysis. Thus, using the Pocock boundary to set the a level, we planned to stop the data collection when 60 participants were collected and conduct our analysis. If the critical test was *p* < .0294, then we would stop our data collection. If not, we would collect the remaining 60 participants.

The final sample included 60 US participants (30 males, 30 females, *M_age_* = 43.88, *SD_age_* = 12.89) collected on Prolific Academic and paid for their participation in a six-minute study. Four screening criteria were applied: participants were English speakers, declaring to live in the United States, with an approval rate of at least 95%, and with at least 100 previous submissions on Prolific. We also exclude participants who previously partook in related studies conducted by our research group.

#### Materials

We asked ChatGPT to generate a list of positive and widely recognized stereotypes related to various social groups.^3^ Among the full list, we selected 40 stereotypes based on nationality, religion, sports, occupation, age, gender, and sexual orientation (e.g., ‘Women are nurturing’, ‘Gay men are fashionable’, ‘Italians are family-oriented’; see Supplemental Materials – Appendix A for the full list of stimuli). To prevent any potential conflicting effects of group repetition, each social group was presented only once. Additionally, we employed 40 distinct traits to represent each social group to avoid any confounding effect of traits repetition throughout the paradigm.

#### Procedure

The experiment was programmed in Inquisit 6. After entering demographic information, participants underwent an exposure phase in which a series of 20 stereotypes appeared individually on the screen. The 20 stereotypes were randomly selected for each participant from a full set of 40 stereotypes. Stereotypes were sequentially presented on the screen for 2000 ms each, with a 1000 ms break between sentences. During the exposure phase, participants were instructed to pay attention to all the statements appearing individually and sequentially on the screen. Next, participants entered the judgment phase. They saw a list of 40 stereotypes, including 20 stereotypes encountered in the exposure phase and 20 stereotypes not encountered. Participants’ task was to rate the extent to which each stereotype was true/false, on a scale from 1 (completely false) to 6 (completely true), in line with recent studies investigating the truth effect (e.g., De keersmaecker et al. 2024; Mattavelli et al. 2023; Mattavelli et al. 2024). Stereotypical statements remained on the screen until participants responded. The next trial was then presented after a break of 1000 ms. Finally, participants were thanked for their participation and debriefed.

### Results

Data were analysed in a paired sample t-test in R (see Supplemental Materials – Appendix C for non-preregistered analyses using mixed-effects linear regression model). We also computed Bayesian factors in accordance with procedures outlined by Rouder et al. (2009) to estimate the amount of evidence for the hypothesis that there was a difference in truth for repeated versus unrepeated stereotypes (alternative hypothesis) or that there is no difference (null hypothesis). We used the default Cauchy prior with a scale of 0.707, which is commonly recommended for t-tests (Rouder et al. 2009).

**Figure 1:**
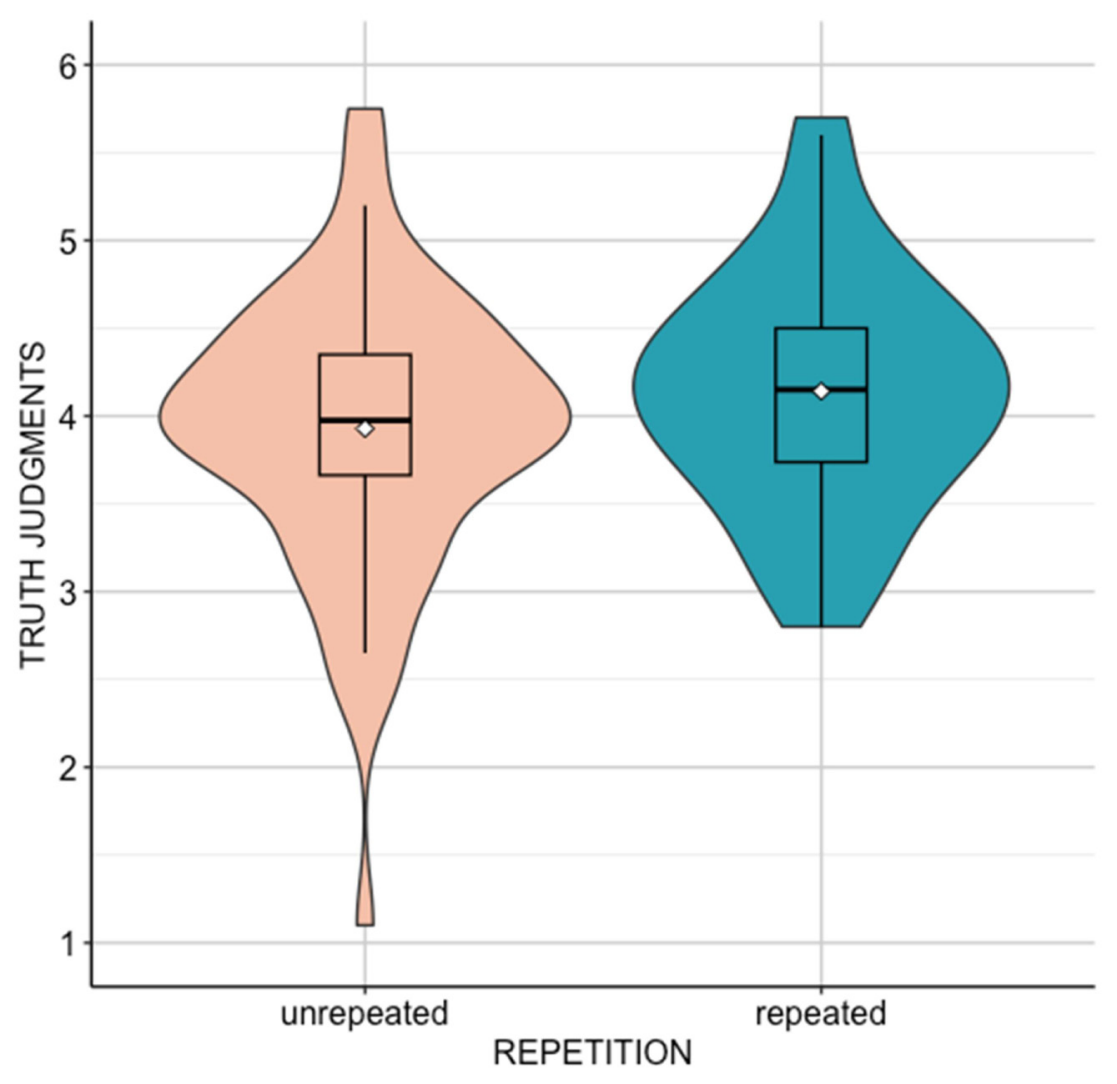
**TViolin plot - Experiment 1.** The boxes are the interquartile range; the bars represent the median; the white dots represent the mean.

Our analysis revealed a significant difference in truth attributed to repeated (*M* = 4.14, *SD* = 0.67) versus unrepeated (*M* = 3.93, *SD* = 0.79) stereotypes, *t*(59) = 2.86, *p* = 0.006, *d_z_* = 0.37 (the correlation between truth judgments given to repeated and unrepeated stereotypes was *r* = 0.70, *p* < 0.001) (see Figure 1). A Bayesian analysis revealed a Bayes factor of BF_10_ = 5.56.

Thus, we found a significant effect of repetition on truth judgments: repeated stereotypes were judged as more true than unrepeated ones. This study showed that exposure to stereotypes is an important variable that contributes to influencing one’s tendency to believe in stereotypes content.

## Experiment 2

Beyond its effect on truth, repetition can also alter the ethical perception of facts and events (Effron 2022; Effron & Raj 2020; Pillai et al. 2023). Positive stereotypes have negative moral consequences (see Czopp et al. 2015). As individuals become attuned to such consequences, the perceived favourability of relying on positive stereotypes for forming impressions about group members may diminish, recasting it as a morally inappropriate action. In this second Experiment our aim was twofold. First, we aimed at replicating the truth effect observed in Experiment 1. Secondly, we investigated whether repetition could also influence the degree to which participants perceived using positive stereotypes to make inferences about members of a targeted group as morally wrong. To address these aims, we employed a between-subjects design with two different dependent variables for each group. Having distinct groups for each dependent variable ensured that participants’ judgments about the truth of stereotypes were not influenced by their moral evaluations, and vice versa. Thus, this separation allows for a better interpretation of the effects of repetition on each dependent variable independently.

### Method

We adopted a one factor (Stereotypes repetition: repeated vs. unrepeated) within-subject ANOVA design and tested the effect on two separate outcome variable – namely, stereotype truth and stereotype wrongness, each measured on two separate groups.

#### Participants

We conducted an a priori power analysis with G*Power 3.1 (Faul et al. 2009). The target effects were the main effect of statements repetition (repeated vs. unrepeated) on (i) truth ratings and (ii) wrongness ratings. Based this analysis, collecting 90 participants in each outcome variable condition (total N = 180) allowed detecting two effects (one tailed paired-sample t-tests) as small as *Cohen’s d* = 0.35 (small to medium), at a = 0.05, and power 1-b = 0.95. Mirroring Experiment 1, we adopted a sequential analysis approach (Lakens 2014). We planned to conduct a single interim analysis. Thus, using the Pocock boundary to set the a level, we stopped the data collection when 90 participants (45 per condition) were collected and conduct our analysis. As the critical tests were not both *p* < 0.0294 after collecting 90 participants, we proceeded by collecting 180 participants.

The final sample included 180 US participants (88 males, 88 females, 4 unspecified, *M_age_* = 46.43, *SD_age_* = 13.23) collected on Prolific Academic and paid for their participation in the six-minute study. We applied the same screening criteria used in Experiment 1 and further excluded participants who partook in that study.

#### Materials

The stimuli were the same as those used in Experiment 1.

#### Procedure

The experiment was programmed in Inquisit 6. After entering demographic information, participants underwent an exposure phase that largely mirrored that used in Experiment 1. Differently from what done in Experiment 1, statements were explicitly presented as stereotypes about social groups. Next, participants were introduced to the judgment phase. In one condition (i.e., truth), participants were asked to evaluate the extent to which each stereotype was true or false. In the other condition (i.e., wrongness), participants were first instructed about the negative moral consequences of forming impressions on group members based on group-stereotypes (see Supplemental Materials – Appendix B for instructions verbatim). Then, participants indicated the extent to which applying each stereotype to new members of the targeted group was morally wrong. For both truth and wrongness judgments, participants rated stereotypes using a scale ranging from 0 (truth: completely false; wrongness: not at all wrong) to 100 (truth: completely true; wrongness: extremely wrong). The choice of a 0–100 scale was based on earlier research on the impact of repetition on both truth and the condemnation of unethical acts (Pillai et al. 2023). This choice also allowed us to test whether the truth effect found in Experiment 1 generalized on a difference response scale. Finally, participants were thanked for their participation and debriefed.

### Results

We tested the effect of statements’ repetition (i.e., unrepeated minus repeated) separately on statements’ truth ratings and wrongness ratings by conducting two separate one-tailed pairwise t-tests (see Supplemental Materials – Appendix C for non-preregistered analyses using mixed-effects linear regression model).

Our analyses replicated the effect of repetition on perception of stereotypes truth, *t*(89) = 2.81, *p* = 0.003, *d_z_* = 0.30 (correlation between truth ratings for repeated and unrepeated stereotypes: *r* = 0.78, *p* < 0.001): Repeated stereotypes were rated as more truthful (*M* = 65.60, *SD* = 12.70) than unrepeated ones (*M* = 63.20, *SD* = 11.40). Bayesian analysis conducted to assess the evidence in favour of the alternative hypothesis (default Cauchy prior with a scale of 0.707) revealed a Bayes factor of BF_10_ = 4.64.

**Figure 2:**
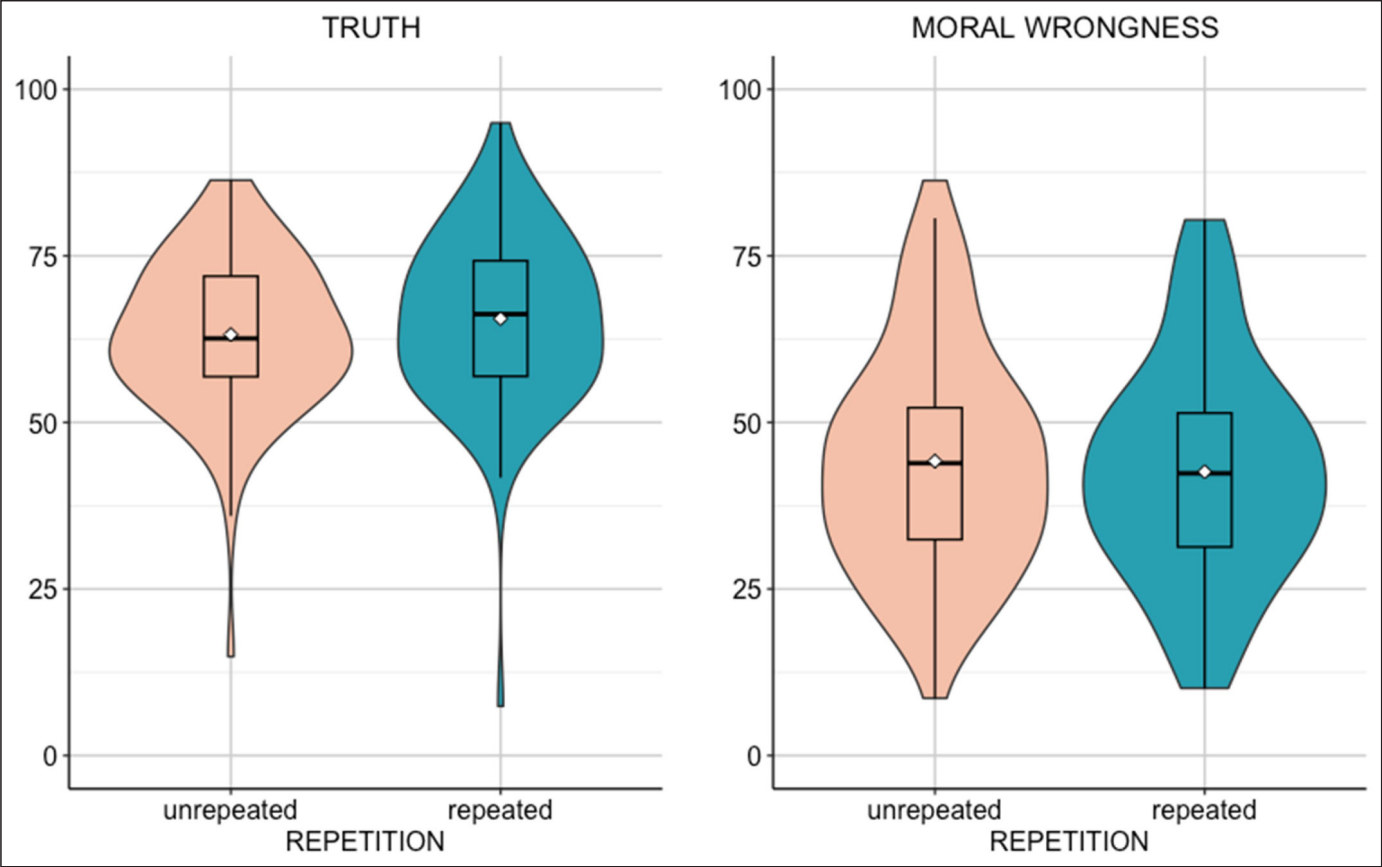
**Violin plots - Experiment 2.** The boxes are the interquartile range; the bars represent the median; the white dots represent the mean.

Moreover, we found a significant effect on judgments of moral wrongness, *t*(89) = –2.54, *p* = 0.006, *d_z_* = –0.27 (correlation between moral wrongness for repeated and unrepeated stereotypes: *r* = 0.89, *p* < 0.001): repeated stereotypes were judged as less immoral (*M* = 42.10, *SD* = 16.80) than unrepeated ones (*M* = 44.20, *SD* = 16.70) (see Figure 2).^4^ Bayesian analysis conducted to assess the evidence in favour of the alternative hypothesis (default Cauchy prior with a scale of 0.707) revealed a Bayes factor of BF_10_ = 2.41.

Thus, Experiment 2 confirmed the effect of repetition on perceived truth of positive stereotypes. Remarkably, this result generalized on a different response scale and with statements explicitly presented as reporting stereotypes about social groups. Central for the present investigation, participants reported that relying on positive stereotypes to form an impression on new members of the target group was less wrong when such stereotypes were repeated than unrepeated.

## General Discussion

What impact does repeated exposure have on positive stereotypes perception? Two pre-registered experiments addressed this question. Participants judged the truth value (Experiments 1–2) and the moral condemnation (Experiment 2) of positive stereotypes, which were manipulated to be either previously encountered or not encountered during an exposure phase. Repeated positive stereotypes were judged as more true than unrepeated ones, demonstrating a truth effect. Additionally, using repeated positive stereotypes to make dispositional attributions about new members of the target group was perceived as less morally wrong than doing the same with newly encountered stereotypes, indicating a moral-repetition effect.

Our findings extend prior research investigating the impact of repetition on positive traits attached to unknown social groups (Oğuz Taşbaş & Unkelbach 2022). We demonstrated that a repetition-induced truth effect persists even when applied to widely recognized beliefs about various social groups, amidst the presence of competing variables such as individuals’ past experiences with the target group. By investigating the truth effect using known stereotypes, we aimed to determine whether a single additional exposure during the experiment could still have an impact beyond the likely prior real-life exposure. In our controlled experimental setting, we deliberately and systematically manipulated the exposure to specific stereotypes to isolate the effect of experimental exposure from participants’ prior exposure in the real world. In acknowledging that experimental exposure may not perfectly mirror real-world exposure to stereotypes, understanding their interplay offers important insights on the relative influence of exposure. Our results suggest that even a single (additional) encounter with already familiar stereotypical statements can significantly enhance their perceived truthfulness. These findings are also relevant to understanding phenomena like illusory correlations (Mullen & Johnson 1990). Illusory correlation occurs when individuals perceive a relationship between two variables (e.g., a social group and a trait) even when no such relationship exists. The truth effect can contribute to the formation and reinforcement of illusory correlations. When individuals repeatedly encounter certain stereotypes, they may begin to perceive a stronger association between the social group and the stereotypical traits. This perception can persist and even be amplified in real-world contexts where these stereotypes are encountered frequently.

While increasing believability, repeating positive stereotypes diminished their moral condemnation, revealing a moral-repetition effect (Effron 2022; Effron & Raj 2020; Pillai et al. 2023). There are different plausible explanations for the negative effect of exposure on the moral condemnation of positive stereotypes. One possibility lies in the mere exposure effect (Zajonc 1968; for reviews, see also Bornstein 1989; Montoya et al. 2017): as repeated information becomes more positive and familiar, people should reduce their moral condemnation of adopting positive stereotypes that are repeated. Going beyond a mere positivity/familiarity effect of repetition, Effron (2022) also argued and demonstrated that the moral repetition effect is mediated by a perceived increase normativity of the repeated immoral act and by a reduced affective intensity in response to it. In other words, people tend to see immoral behaviours as more frequent and normal when they are repeated and react with lower anger to them (see also Pillai et al. 2023).

Additionally, the increased perceived truth due to repetition might also contribute to explain the moral repetition effect. In Experiment 2, truth and moral condemnation were kept separate and measured on two different groups of participants. This was done to prevent responses to one variable from influencing responses to the other. However, exploring the causal path from exposure to truth perception and from truth to moral condemnation could provide valuable insights for the understanding of the moral repetition effect with positive stereotypes. For instance, repeated exposure might generate consensus about positive stereotypes and justify their diffusion among the general population, despite their inaccuracy. Consistently with this idea, Effron (2022) showed that repeated exposure to fake-news increased one’s tendency to share them. Likewise, as individuals exposed to positive stereotypes tend to perceive them as more truthful and morally acceptable, they may be more inclined to propagate such beliefs within their social circles. This would ultimately lead to a self-perpetuating cycle; in other words, individual exposure increases the believability of positive stereotypes and reduces moral condemnation that can foster collective exposure. Future studies should test the role of these potential mediating variables (i.e., positivity, normativity, affective intensity, and truth) on the moral repetition effect with stereotypes.

Importantly, our work focused solely on the role of exposure in altering the perception of positive stereotypes. Whether these findings are generalizable to negative stereotypes about social groups remains an empirical question for future studies to address. Regarding the truth effect, recent research has indicated that repetition is more likely to increase perceived truth for positive, as opposed to negative, information about target individuals (Mattavelli et al. 2024). However, Mattavelli et al. (2024) studied the impact of valence on the truth effect by considering statements about unknown individuals. Although we are not aware of any study investigating how valence could moderate the truth effect on stereotypes, it seems plausible that negative information about social groups might elicit different cognitive responses compared to positive information (Unkelbach et al. 2019), leading to inconsistent effects of repetition. For instance, while repeating positive information about a group (e.g., ‘women are caring’) might be generally perceived as a benevolent act (Czopp et al. 2015), repeating negative information (e.g., ‘women are bad at math’) typically fosters interpersonal mistrust. In the latter case, people may need more substantial justification beyond mere repetition to believe the negative information about the target group.

Regarding the moral repetition effect, despite using positive stereotypical statements in our study, participants were instructed about the negative moral consequences of forming impressions of group members based on stereotypes, including positive ones. This was done to emphasize the moral wrongness of using positive stereotypes when judging social groups. Although the average moral wrongness attributed to endorsing positive stereotypes was overall moderate (slightly below the scale mid-point), participants must have believed that endorsing positive stereotypes qualifies as a ‘somewhat immoral’ act. Assuming that (a) the moral wrongness attributed to using negative stereotypes would increase and that (b) past research indicates that the moral repetition effect emerges robustly on behaviours that are judged as highly immoral at their baseline (average value higher than 70 on a 0–100 immorality scale, see Effron 2022), then the effect of repetition on reducing the moral condemnation of stereotypes should emerge also on negative stereotypes. Notably, finding a moral repetition effect result with participants being explicitly informed about the negative moral consequences of relying on stereotypical beliefs might raise concern on the ecologic validity of our paradigm. Namely, in real-world scenarios, individuals might not receive such explicit information, and the moral condemnation of positive stereotypes may be influenced by other factors. Replicating the study without making the negative consequences salient can offer insights into how the effect of repeated exposure operates under more naturalistic conditions.

Finding a truth effect and moral-repetition effect in the context of stereotypes does not imply that exposure is the only variable accounting for the negative consequences of stereotypes. We acknowledge that the magnitude of the observed effects qualifies as small-to-medium on both truth (*d*z = 0.37 and *d*z = 0.30) and moral wrongness (*dz* = –0.27). Yet, because stereotypes perception is a complex phenomenon, reliably estimating even small effects remains pivotal (Götz et al. 2022). Our research highlights the importance of considering exposure as a factor that can amplify the negative consequences of positive stereotypes. However, our investigation limits to the impact of exposure on the short run. Our experiments were designed to assess immediate reactions following exposure to positive stereotypes. Whereas this approach allowed us to measure cognitive and emotional responses triggered by positive stereotype repetition, our findings do not necessarily reflect the long-term effects of exposure. Future research should investigate the persistence and potential escalation of these effects over longer periods.

Finally, unravelling the role of exposure in stereotype perception holds significant implications for intervention strategies aimed at fighting stereotypes. Despite the widespread impact of repetition, recent findings showed that contrasting the truth effect is possible (e.g., Brashier et al. 2020; Mattavelli et al. 2023; Nadarevic & Aßfalg 2017). Brashier et al. (2020) showed that asking participants to focus on information accuracy eliminated the truth effect. As an alternative strategy, Mattavelli et al. (2023) found that presenting unknown information in an interrogative form largely reduces the truth effect. These findings offer promising avenues for mitigating the negative effects of exposure on stereotypes perception.

In conclusion, our study sheds light on the role of exposure on the perception of positive stereotypes. Increased exposure not only strengthens the belief in their truth but also reduces the moral condemnation associated with endorsing them.

## Additional File

The additional file for this article can be found as follows:

10.5334/irsp.933.s1 Supplementary Materials. Appendix A, B and C.

## Footnotes

Experiment 1: https://osf.io/5mu8q/; Experiment 2: https://osf.io/4p62x/.

Experiment 1: https://osf.io/qkbfv/; Experiment 2: https://osf.io/kda3v/.

We used the GPT-4 version of ChatGPT. We ask ChatGPT to generate recognized positive stereotypes for each of the following categories: (a) gender, (b) ethnicity, (c) nationality, (d) religion, (e) sexual orientation, (f) age, (g) job, (h) political affiliation, and (i) sport.

We also tested the interaction between statements repetition and measure using the ezANOVA () function in R. The analysis revealed a main effect of measure, *F*(1, 178) = 102.61, *p* < 0.001, η^2^_G_ = 0.348, no significant effect of statements repetition, *F*(1, 178) = 0.08, *p* = 0.774, η^2^_G_ < 0.001, and a significant interaction, *F*(1, 178) = 14.37, *p* < 0.001, η^2^_G_ = 0.006.
