# Supplementary material for: True and Moral by Repetition: Unveiling the Impact of Exposure on Positive Stereotypes Perception
Source: Int Rev Soc Psychol. 2024 Aug 7;37:14. doi: 10.5334/irsp.933 (PMC12372772; doi:10.5334/irsp.933)
Supplement: Supplementary Materials. — Appendix A, B and C. [file irsp-37-933-s1.pdf]

Supplemental Materials

Appendix A. Positive stereotypes (in alphabetical order)

1. Americans are outgoing
2. Artists are unconventional
3. Asian people are intelligent
4. Australians are adventurous
5. Black people are musical
6. Brazilians are passionate
7. British people are polite
8. Buddhists are peaceful
9. Canadians are friendly
10. Chinese are hardworking
11. Christians are charitable
12. Doctors are wealthy
13. Engineers are logical
14. French people are sophisticated
15. Gay men are fashionable
16. Germans are efficient
17. Greeks are hospitable
18. Hawaiians are laid-back
19. Indians are spiritual
20. Irish people are warm
21. Italians are family-oriented
22. Japanese are disciplined
23. Jews are intellectual
24. Lawyers are ambitious
25. Lesbians are empowered
26. Men are protective
27. Mexicans are fun-loving
28. Musicians are creative
29. Muslims have strong sense of community
30. Nigerians are resilient
31. Older adults are wise
32. Queer individuals are nonconformist
33. Russians are stoic
34. Salespeople are smooth talkers
35. Swimmers are dedicated
36. Swiss people are precise
37. Teachers are caring
38. Tennis players are elegant
39. Women are nurturing
40. Young people are tech-savvy

Appendix B. Instructions verbatim

Experiment 1

Presentation phase

Page 1. “Dear Participant, you are participating in a study investigating various influences on people's assessment of sentences’ content.”

Page 2. “In the first part, you will only see a presentation of 20 sentences about different social groups.

Please try to read all sentences, even though the presentation is fast. If you have no further questions, please commence with the presentation”

Judgment phase

Page 1. “The evaluation phase is now going to start.

We are going to present you 40 sentences about social groups.

The content of some sentences has been presented to you in the previous phase, whilst the content of other sentences will be new.”

Page 2. “Your job is to rate the content of each sentence on a scale from 1=completely false to 6=completely true.”

Experiment 2

Presentation phase

Page 1. “Dear Participant, you are participating in a study investigating various influences on people's assessment of sentences’ content.”

Page 2. “In the first part, you will only see a presentation of 20 sentences reporting stereotypes about different social groups.

Please try to read all sentences, even though the presentation is fast. If you have no further questions, please commence with the presentation”

Judgment phase: *Truth*

Page 1. “The evaluation phase is now going to start.

We are going to present you 40 sentences reporting stereotypes about social groups.

The content of some sentences has been presented to you in the previous phase, whilst the content of other sentences will be new.”

Page 2. “In evaluation phase, your job is to rate the content of each sentence on truth, using a a scale ranging from completely false (0) to completely true (100).”

Judgment phase: *Wrongness*

Page 1. “The evaluation phase is now going to start.

We are going to present you 40 sentences reporting stereotypes about social groups.

The content of some sentences has been presented to you in the previous phase, whilst the content of other sentences will be new.”

Page 2. “Stereotypes are often used as shortcuts to make attribution on new members of a target group.

However, relying on stereotypes does not come without moral consequences.

The moral issue with stereotypes arises when they are used inappropriately or without considering individual differences. Stereotyping can lead to unfair judgments, discrimination, prejudice, and perpetuation of harmful biases."

Page 3. "In this phase, your job is to assess the moral implications of applying each stereotype universally to any member of the relevant group.

Please assess the moral implications using a scale ranging from 0 (not at all wrong) to 100 (extremely wrong)."

Appendix C. Non-preregistered analyses with mixed-effects linear regression.

Experiment 1

We conducted a mixed-effects linear regression model predicting truth ratings from repetition (i.e., repeated vs unrepeated statements) with random intercepts by participants and stereotypes and repetition slopes by participants. Before fitting the model, we passed it through the buildmer package in R (Voeten, 2019). The model converged, but if it had not, then buildmer would have systematically removed random effects until it achieved convergence. Results showed that participants rated repeated stereotypes as significantly as more truthful than unrepeated stereotypes (repeated: $M = 4.14$, $SD = 1.14$; unrepeated: M = 3.93.85, $SD = 1.19$), $b = 0.21$, $SE = 0.07$, $t(58.93) = 2.97$, $p = .004$, $dz = 0.38$.

Experiment 2

We determined the most complex models for the effect of repetition on both truth and moral wrongness. Both the models included repetition as fixed effect (i.e., repeated vs unrepeated statements) with random intercepts by participants and stereotypes and repetition slopes by participants.

Our analysis replicated the effect of repetition on truth: participants rated repeated stereotypes as significantly as more truthful than unrepeated stereotypes (repeated: $M = 65.60$, $SD = 21.20$; unrepeated: $M = 63.20$, $SD = 21.60$; $dz = 0.38$), $b = 2.83$, $SE = 0.78$, $t(88.77) = 3.64$, $p < .001$.

Moreover, we found a significant effect of repetition on judgments of moral wrongness, revealing that using repeated stereotypes was judged as less morally wrong than using unrepeated ones (repeated: $M = 42.10$, $SD = 27.60$; unrepeated: $M = 44.20$, $SD = 27.80$; $dz = -0.23$), $b = -1.66$, $SE = 0.76$, $t(89.01) = -2.20$, $p = .031$.
